# On the least signless Laplacian eigenvalue of a non-bipartite connected graph with fixed maximum degree

**DOI:** 10.1186/s13660-017-1395-y

**Published:** 2017-05-22

**Authors:** Shu-Guang Guo, Rong Zhang

**Affiliations:** School of Mathematics and Statistics, Yancheng Teachers University, Yancheng, Jiangsu 224002 P.R. China

**Keywords:** 05C50, non-bipartite graph, signless Laplacian, least eigenvalue, maximum degree

## Abstract

In this paper, we determine the unique graph whose least signless Laplacian eigenvalue attains the minimum among all non-bipartite unicyclic graphs of order *n* with maximum degree Δ and among all non-bipartite connected graphs of order *n* with maximum degree Δ, respectively.

## Introduction

All graphs considered in this paper are finite, simple and undirected. Let *G* be a graph with vertex set $V=V(G)= \{v_{1}, v _{2}, \ldots, v_{n}\}$ and edge set $E=E(G)$. Write $A(G)$ for the adjacency matrix of *G* and let $D(G)$ be the diagonal matrix of the degrees of *G*. The matrix $Q(G)=D(G)+ A(G)$ is called the signless Laplacian matrix of *G*. As usual, let $q_{1}(G)\ge q_{2}(G) \ge \cdots\ge q_{n}(G)\ge0$ denote the eigenvalues of $Q(G)$ and call them the signless Laplacian eigenvalues of *G*. Denote by $\kappa(G)$ the least eigenvalue of *G*.

For a connected graph *G*, $\kappa(G) = 0$ if and only if *G* is bipartite. Desai and Rao [1] suggest the use of $\kappa(G)$ as a measure of non-bipartiteness of *G*. Fallat and Fan [2] introduce two parameters reflecting the graph bipartiteness, and establish a relationship between $\kappa(G)$ and the two parameters. de Lima, Nikiforov and Oliveira [3] point out that $\kappa(G)$ depends more on the distribution of the edges of a graph than on their number, so it may become a useful tool in extremal graph theory. *For a connected non-bipartite graph* *G* *with given order, how small can* $\kappa(G)$ *be?* Cardoso *et al.* [4] propose this problem and show that the minimum value of $\kappa(G)$ is attained uniquely in the unicyclic graph obtained from the cycle $C_{3}$ by attaching a path at one of its end vertices. Motivated by this problem, a good deal of attention has been devoted to finding all graphs with the minimal least signless Laplacian eigenvalue among a given class of graphs. For related results, one may refer to [5–14].

A unicyclic graph is a connected graph with a unique cycle. Let $\Delta=\Delta(G)$ be the maximum degree of a graph *G*. In this paper, we determine the unique graph whose least signless Laplacian eigenvalue attains the minimum among all non-bipartite unicyclic graphs of order *n* with maximum degree Δ and among all non-bipartite connected graphs of order *n* with maximum degree Δ, respectively.

The rest of the paper is organized as follows. In Section 2, we recall some notions and lemmas used further, and prove three new lemmas. In Section 3, we prove two theorems which is our main result. In Section 4, we propose two problems for further research.

## Preliminaries

Denote by $C_{n}$ the cycle on *n* vertices. Let $G-uv$ denote the graph which arises from *G* by deleting the edge $uv\in E(G)$. Similarly, $G+uv$ is the graph that arises from *G* by adding an edge $uv\notin E(G)$, where $u, v\in V(G)$. For $v\in V(G)$, $N(v)$ denotes the neighborhood of *v* in *G* and $d(v)=\vert N(v)\vert $ denotes the degree of vertex *v*. A pendant vertex of *G* is a vertex of degree 1. $\vert x\vert $ denotes the absolute value of a real number *x*. The terminology not defined here can be found in [15].

### Lemma 2.1

[16]

*Let* *G* *be a graph on* *n* *vertices*, *e* *be an edge of* *G*. *Then*

$$q_{1}(G)\ge q_{1}(G-e)\ge q_{2}(G)\ge q_{2}(G-e)\ge\cdots\ge q_{n}(G) \ge q_{n}(G-e)\ge0. $$

Given $x=(x_{1}, x_{2}, \ldots, x_{n})^{T} \in R^{n}$, we can define a function on $V(G)$, that is, each vertex $v_{i}$ is mapped to $x_{i} =x(v_{i})$. If *x* is an eigenvector of $Q(G)$, then it is defined on *G* naturally, *i.e.* $x(v)$ is the entry of *x* corresponding to *v*. Clearly, for $x\in R^{n}$,

$$x^{T}Q(G)x =\sum_{uv\in E(G)}\bigl(x(u) + x(v) \bigr)^{2}. $$

Let $x\in R^{n}$ be an arbitrary unit vector. One can find in [10, 15] that

1 $$ \kappa(G) \le x^{T}Q(G)x, $$

with equality if and only if *x* is an eigenvector corresponding to $\kappa(G)$.

Let $G_{1}$ and $G_{2}$ be two vertex-disjoint connected graphs, and let $v_{i}\in V(G_{i})$ for $i=1, 2$. Identifying the $v_{1}$ with $v_{2}$ and forming a new vertex *u* (see [10] for details), the resulting graph is called coalescence of $G_{1}$ and $G_{2}$, and denoted by $G_{1}(v_{1})\diamond G_{2}(v_{2})$ or $G_{1}(u)\diamond G _{2}(u)$. If a connected graph *G* can be expressed in the form $G_{1}(u)\diamond G_{2}(u)$, where $G_{1}$ and $G_{2}$ are both nontrivial and connected, then $G_{1}$ is called a branch of *G* with root *u*. Clearly $G_{2}$ is also a branch of *G* with root *u*. Let $x\in R^{n}$ be a vector defined on $V(G)$. A branch $G_{i}$ of *G* is called a zero branch with respect to *x* if $x(v) = 0$ for all $v \in V(G_{i})$; otherwise it is called a nonzero branch with respect to *x*.

### Lemma 2.2

[10]

*Let* *G* *be a connected graph which contains a bipartite branch* *B* *with root* *u*, *and* *x* *be an eigenvector corresponding to* $\kappa(G)$.

(i) *If* $x(u) = 0$, *then* *B* *is a zero branch of G with respect to* *x*.
(ii) *If* $x(u)\neq0$, *then* $x(v)\neq0$ *for every vertex* $v\in V(B)$.

### Lemma 2.3

[10]

*Let* *G* *be a non*-*bipartite connected graph*, *and let* *x* *be an eigenvector corresponding to* $\kappa(G)$. *Let* *T* *be a tree*, *which is a nonzero branch of* *G* *with respect to* *x* *and with root* *u*. *Then* $\vert x(q)\vert < \vert x(p)\vert $ *whenever* *p*, *q* *are vertices of* *T* *such that* *q* *lies on the unique path from* *u* *to* *p*.

### Lemma 2.4

[12]

**Figure 1:**
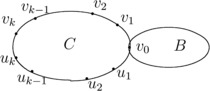
$\pmb{C(v_{0})\diamond B(v_{0})}$ **.**

*Let* $G = C(v_{0})\diamond B(v_{0})$ (*see Figure* 1), *where* $C=v_{0}v _{1}v_{2}\cdots v_{k}u_{k}u_{k-1} \cdots u_{1}v _{0}$ *is a cycle of length* $2k+1$ *and* *B* *is a nontrivial connected bipartite graph*. *Let* $x=( x(v_{0}),x(v_{1}),x(v_{2}),\ldots,x(v_{k}),x(u_{1}), x(u_{2}),\ldots,x(u_{k}),\ldots)^{T}$ *be an eigenvector corresponding to* $\kappa(G)$. *Then*

(i) $\vert x(v_{0})\vert =\max\{\vert x(w)\vert \vert w\in V(C)\}>0$;
(ii) $x(v_{i})=x(u_{i})$ *for* $i=1, 2, \ldots, k$.

### Lemma 2.5

[12]

*Let* $G = G_{1}(v_{2}) \diamond T(u)$ *and* $G^{*} = G_{1}(v_{1})\diamond T(u)$, *where* $G_{1}$ *is a non*-*bipartite connected graph containing two distinct vertices* $v_{1}$, $v_{2}$, *and* *T* *is a nontrivial tree*. *If there exists an eigenvector* $x=( x(v_{1}),x(v_{2}),\ldots,x(v_{k}),\ldots)^{T}$ *corresponding to* $\kappa(G)$ *such that* $\vert x(v_{1})\vert > \vert x(v_{2})\vert $ *or* $\vert x(v_{1})\vert = \vert x(v_{2})\vert > 0$, *then* $\kappa(G^{*})<\kappa(G)$.

For $k \ge1$, let $G'$ denote the graph obtained from *G* by deleting the edge *uv*, inserting *k* new vertices $v_{1}, v_{2}, \ldots, v _{k}$ and adding edges $uv_{1}, v_{1}v_{2}, \ldots, v_{k-1}v_{k}, v _{k}v$. Then $G'$ is called a *k*-subdivision graph of *G* by *k*-subdividing the edge *uv*.

### Lemma 2.6

[17]

*Let* $G'$ *be a* *k*-*subdivision graph of a graph* *G*. *If* *k* *is even*, *then* $\kappa(G')\le\kappa(G)$.

**Figure 2:**
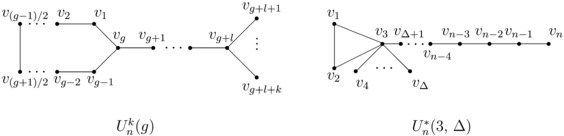
$\pmb{U_{n}^{k}(g)}$ **and** $\pmb{U_{n}^{*}(3, \Delta)}$ **.**

$U_{n}^{k}(g)$, showed in Figure 2, denotes the unicyclic graph on *n* vertices with odd girth *g* and *k* pendant vertices, where $g+l+k=n$. $U_{n}^{*}(3, \Delta)$, showed in Figure 2, denotes the unicyclic graph on *n* vertices obtained from the cycle $C_{3}=v_{1}v _{2}v_{3}v_{1}$ by attaching $\Delta-3$ pendant edges and one pendant path at the vertex $v_{3}$.

### Lemma 2.7

[5, 9]

*Among all non*-*bipartite connected graphs on* *n* *vertices with* *k* *pendant vertices*, $U_{n}^{k}(3)$ *is the unique graph whose signless Laplacian eigenvalue attains the minimum*.

### Lemma 2.8

[5]

*Let* $k\ge2$, *and* $g\ge3$ *be an odd integer*. *Then* $\kappa(U_{n}^{k-1}(g))< \kappa(U_{n}^{k}(g))$.

### Lemma 2.9

*Let* $G = G_{1}(v)\diamond B(v)$ *be a connected graph*, *where* $G_{1}$ *is a graph of order* *n*, *and* *B* *is a bipartite graph of order* *s*. *Then* $\kappa(G ) \le\kappa(G_{1})$. *Moreover*, *if* $s>1$, $G_{1}$ *is non*-*bipartite and there exists an eigenvector* *x* *corresponding to* $\kappa(G_{1})$ *such that* $x(v)\neq0$, *then* $\kappa(G )<\kappa(G_{1})$.

### Proof

Let $V(G_{1})=\{ v_{1}, v_{2}, \ldots, v_{n} \}$, and $x=(x(v_{1}), x(v_{2}), \ldots, x(v_{n}))^{T}$ be a unit eigenvector corresponding to $\kappa(G_{1})$. Then

$$\kappa(G_{1})=\sum_{v_{i}v_{j}\in E(G_{1})} \bigl(x(v_{i})+x(v_{j})\bigr)^{2}. $$

Without loss generality, we may assume $v=v_{n}$. Let $V(B)=\{ v_{n}, v_{n+1}, \ldots, v_{n+s-1} \}$, and let $(U, W)$ be the two parts of the bipartite graph *B*, where $v\in U$. Let $y=(y(v_{1}), y(v_{2}), \ldots, y(v_{n}), y(v_{n+1}), \ldots, y(v_{n+s-1}))^{T}\in R^{n+s-1}$ defined on $V(G)$ satisfy that $y(v_{i})=x(v_{i})$ for $i=1, 2, \ldots, n$, $y(u)=x(v)$ if $u\in U$, and $y(u)=-x(v)$ if $u\in W$. Then

$$\begin{aligned}& \Vert y\Vert ^{2}=\sum_{i=1}^{n+s-1}y(v_{i})^{2}= \sum_{i=1}^{n}x(v_{i})^{2}+(s-1)x(v)^{2} \ge\sum_{i=1}^{n}x(v_{i})^{2}= \Vert x\Vert ^{2}=1, \\& \kappa\bigl(G^{*}\bigr)\le\frac{1}{\Vert y\Vert ^{2}} \sum _{v_{i}v_{j}\in E(G^{*})}\bigl(y(v _{i})+y(v_{j}) \bigr)^{2}\le\frac{1}{\Vert x\Vert ^{2}}\sum_{v_{i}v_{j}\in E(G)} \bigl(x(v _{i})+x(v_{j})\bigr)^{2}=\kappa(G). \end{aligned}$$

Clearly, if $s>1$, $G_{1}$ is non-bipartite and $x(v)\neq0$, we have $\Vert y\Vert ^{2}>\Vert x\Vert ^{2}$. This implies that $\kappa(G) <\kappa(G_{1})$. □

### Lemma 2.10

**Figure 3:**
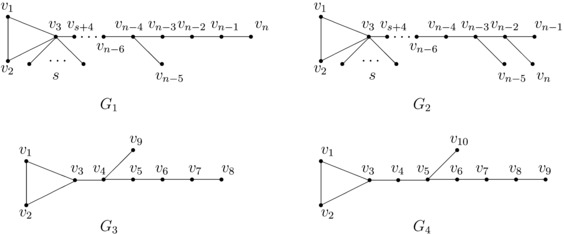
$\pmb{G_{1}-G_{4}}$ **.**

*Let* $n\ge9$ *and* $s\ge0$ *be integer*. $G_{1}$ *and* $G_{2}$, *shown in Figure* 3, *are two unicyclic graphs of order* *n*. *Then* $\kappa(G_{2})< \kappa(G_{1})$.

### Proof

Let $\kappa=\kappa(G_{1})$, and $x=(x_{1}, x_{2}, \ldots, x_{n})^{T}$ be a unit eigenvector corresponding to *κ*. Then $\kappa= \sum_{v_{i}v_{j}\in E(G_{1})}(x_{i}+x_{j})^{2}$ and $0<\kappa<1$. By Lemmas 2.2 and 2.4, we have $x_{n}\neq0$. From the eigenvalue equation $Q(G_{1})x =\kappa x$, we have

$$\begin{aligned}& x_{n-1} =(\kappa-1)x_{n}, \\& x_{n-2} =\bigl(\kappa^{2}-3\kappa+1\bigr)x_{n}, \\& x_{n-3} =\bigl(\kappa^{3}-5\kappa^{2}+6\kappa-1 \bigr)x_{n}, \\& x_{n-4} =\bigl(\kappa^{4}-7\kappa^{3}+15 \kappa^{2}-10\kappa+1\bigr)x_{n}, \\& x_{n-5} =\bigl(\kappa^{3}-6\kappa^{2}+9\kappa-1 \bigr)x_{n}. \end{aligned}$$

Let $y=(y_{1}, y_{2}, \ldots, y_{n})^{T}\in R^{n}$ defined on $V(G_{2})$ satisfy that

$$\begin{aligned}& y_{n-5}=-(x_{n-3}+x_{n-4}+x_{n-5}),\\& y_{n}=-(x_{n}+x_{n-1}+x_{n-2}), \end{aligned}$$

and $y_{i}=x_{i}$ for $i=1, 2, \ldots, n-6, n-4, n-3, n-2, n-1$. Then

$$\sum_{v_{i}v_{j} \in E(G_{2})}(y_{i}+y_{j})^{2}= \sum_{v_{i}v_{j} \in E(G_{1})}(x_{i}+x_{j})^{2}= \kappa, $$

and

$$\begin{aligned} \Vert y\Vert ^{2}-\Vert x\Vert ^{2} =&\sum _{i=1}^{n} y_{i}^{2}-\sum _{i=1}^{n} x_{i}^{2} \\ =& \kappa\bigl(\kappa^{7}-10\kappa^{6}+32\kappa^{5}-18 \kappa^{4}-89\kappa ^{3}+156\kappa^{2}-70\kappa+4 \bigr)x_{n}^{2}. \end{aligned}$$

Let $f(t)=t^{7}-10t^{6}+32t^{5}-18t^{4}-89t^{3}+156t^{2}-70t+4$. By a computation, $f(t)=0$ has five real roots which are approximately equal to −1.7787, 0.0667, 0.6606, 2, 2.0890, respectively. By Lemma 2.9, we have

$$\kappa=\kappa(G_{1})\le\kappa(G_{1}-v_{4}- \cdots-v_{s+3}). $$

Note that $G_{1}-v_{4}-\cdots-v_{s+3}$ is a 2*t*-subdividing graph of $G_{3}$ or $G_{4}$ (shown in Figure 3). By Lemma 2.6, we have

$$\kappa=\kappa(G_{1})\le\kappa(G_{1}-v_{4}- \cdots-v_{s+3})\le \min\bigl\{ \kappa(G_{3}), \kappa(G_{4}) \bigr\} . $$

By a computation, we have $\kappa(G_{3})\approx0.0588025$ and $\kappa(G_{5})\approx0.0426304$. It follows that $\kappa< 0.0667$. Noting that $f(0)=4$, we have $f(\kappa)>0$. It follows that $\Vert y\Vert ^{2}>\Vert x\Vert ^{2}$.

Combining the above arguments, we have

$$\kappa(G_{2}) \le\frac{1}{\Vert y\Vert ^{2}}\sum_{v_{i}v_{j} \in E(G_{2})}(y _{i}+y_{j})^{2}< \frac{1}{\Vert x\Vert ^{2}}\sum _{v_{i}v_{j} \in E(G_{1})}(x _{i}+x_{j})^{2}= \kappa(G_{1}). $$

This completes the proof. □

### Lemma 2.11

*Let* $n\ge9$, *and* $U_{n}^{n-5}(3)$, $U_{n}^{n-4}(3)$, $U_{n}^{*}(3, n-4)$, $U_{n}^{*}(3, n-3)$ *be shown in Figure* 2. *Then*

$$\kappa\bigl(U_{n}^{n-5}(3)\bigr)< \kappa\bigl(U_{n}^{*}(3, n-4)\bigr),\qquad \kappa \bigl(U_{n}^{n-4}(3)\bigr)< \kappa \bigl(U_{n}^{*}(3, n-3)\bigr). $$

### Proof

Let $\kappa=\kappa(U_{n}^{*}(3, n-4))$, and $x=(x_{1}, x_{2}, \ldots, x_{n})^{T}$ be a unit eigenvector corresponding to *κ*. By Corollary 1.3 of [18], it is easy to see $\kappa(G)<1/2$. From the eigenvalue equation $Q(U_{n}^{*}(3, n-4))x =\kappa x$, we have $x_{1}=x_{2}$, $x_{4}=\cdots=x_{n-4}$,

$$\begin{aligned}& (\kappa-2)x_{1} =x_{1}+x_{3}, \\& (\kappa-n+4) x_{3} =2x_{1}+(n-7)x_{4}+x_{n-3}, \\& (\kappa-1) x_{4} =x_{3}, \\& (\kappa-2)x_{n-3} =x_{3}+x_{n-2}, \\& (\kappa-2)x_{n-2} =x_{n-3}+x_{n-1}, \\& (\kappa-2)x_{n-1} =x_{n-2}+x_{n}, \\& (\kappa-1)x_{n} =x_{n-1}. \end{aligned}$$

Since $x=(x_{1}, x_{2}, \ldots, x_{n})^{T}$ is an eigenvector, $x\neq0$. It follows that

$$\left| \begin{matrix} \kappa-3 & -1 & 0& 0& 0& 0& 0 \\ -2 & \kappa-n+4 &7-n & -1& 0& 0& 0 \\ 0 & -1 & \kappa-1 & 0& 0 & 0& 0 \\ 0 & -1 & 0 & \kappa-2& -1& 0& 0 \\ 0 &0 & 0 & -1&\kappa-2& -1& 0 \\ 0 &0 & 0 & 0&-1&\kappa-2& -1 \\ 0 &0 & 0 & 0&0& -1& \kappa-1 \end{matrix} \right| =0. $$

This implies that *κ* is the least root of the following equation:

$$\begin{aligned} f(x) \triangleq& x^{7}-(n+7)x^{6}+(10n+6)x^{5}-(36n-48)x^{4}+(55n-99)x ^{3} \\ &{}-(31n-15)x^{2}+(3n+40)x-4=0. \end{aligned}$$

Similarly, we can see that $\kappa(U_{n}^{n-5}(3))$ is the least root of the following equation:

$$g(x)\triangleq x^{5}-(n+5)x^{4}+(8n-6)x^{3}-(18n-42)x^{2}+(11n-28)x-4=0. $$

Noting that $g(0)=-4<0$ and

$$f(x)-(x-1)^{2}g(x)=x(x-1) \bigl(x^{3} - nx^{2}- (n-19)x + 8n - 60\bigr)< 0 $$

for $0< x<1/2$, we have $g(\kappa)>0$, and so

$$\kappa\bigl(U_{n}^{n-5}(3)\bigr)< \kappa=\kappa \bigl(U_{n}^{*}(3, n-4)\bigr). $$

By a similar reasoning to above, we can see that $\kappa(U_{n}^{*}(3, n-3))$ and $\kappa(U_{n}^{n-4}(3))$ are the least root of the following equations respectively:

$$\begin{aligned}& h(x)\triangleq x^{6}-(n+6)x^{5}+(8n+5)x^{4}-(21n-18)x^{3}+(19n-10)x ^{2}-(3n+24)x+4=0, \\& r(x)\triangleq x^{4}-(n+4)x^{3}+(6n-5)x^{2}-(7n-12)x+4=0. \end{aligned}$$

Noting that $r(0)=4>0$ and

$$h(x)-(x-1)^{2}r(x)=x\bigl(x^{3} - nx^{2} + (n-15)x + 4n - 28\bigr)>0 $$

for $0< x<1/2$, we have $r(\kappa(U_{n}^{*}(3, n-3)))<0$, and so

$$\kappa\bigl(U_{n}^{n-4}(3)\bigr)< \kappa\bigl(U_{n}^{*}(3, n-3)\bigr). $$

This completes the proof. □

## Main results

Let $\mathcal{U}(n, \Delta)$ be the set of non-bipartite unicyclic graphs of order *n* with maximum degree Δ, and $\mathcal{G}(n, \Delta)$ be the set of non-bipartite connected graphs of order *n* with maximum degree Δ. In this section, we firstly determine the unicyclic graph whose signless Laplacian eigenvalue attains the minimum among all graphs in $\mathcal{U}(n, \Delta)$.

### Theorem 3.1

*Let* $4\le\Delta\le n-3$. *Among all graphs in* $\mathcal{U}(n, \Delta)$, $U_{n}^{ \Delta-1}(3)$ *is the unique graph whose signless Laplacian eigenvalue attains the minimum*.

### Proof

Let $G\in\mathcal{U}(n, \Delta)$, and $C_{g}=v_{1}v_{2}\ldots v _{g}v_{1}$ be the unique cycle of *G*. Then *g* is odd, and *G* can be obtained by attaching trees $T_{1},T_{2},\ldots,T_{g}$ to the vertices $v_{1},v_{2},\ldots,v_{g}$ of $C_{g}$, respectively, where $T_{i}$ contains the root vertex $v_{i}$ for $i=1,2, \ldots, g$. $\vert V(T_{i})\vert =1$ means $V(T_{i})=\{v_{i}\}$. Suppose that *G* has *k* pendant vertices. It is easy to see $\Delta\le k+2$. Let $x=(x_{1}, x _{2}, \ldots, x_{n})^{T}$ be a unit eigenvector corresponding to $\kappa(G)$.

*Case* 1. $\Delta\le k+1$. By Lemma 2.7, we have $\kappa(U_{n}^{ k}(3))\le\kappa(G)$ with equality if and only if $G=U_{n}^{ k}(3)$. By Lemma 2.8, we have $\kappa(U_{n}^{ \Delta-1}(3))\le\kappa(U_{n}^{ k}(3))$ with equality if and only if $\Delta=k+1$. It follows that $\kappa(U_{n}^{ \Delta-1}(3))\le \kappa(G)$ with equality if and only if $G=U_{n}^{ \Delta-1}(3)$.

*Case* 2. $\Delta=k+2$. Then *G* must be the graph obtained from the cycle $C_{g}$ with *k* pendant paths $P_{i_{1}},\ldots,P_{i_{k}}$ attached at the same vertex $v_{1}$ of $C_{g}$, and $k\ge2$.

If $g\ge5$, by Lemma 2.4, we have $x_{(g-3)/2}=x_{(g+3)/2}$ and $\vert x_{2}\vert \le \vert x_{1}\vert $. Let

$$G '=G-v_{(g-1)/2}v_{(g-3)/2}+v_{(g-1)/2}v_{(g+3)/2}. $$

Then $\Delta(G ')=\Delta$, $G '$ has $k+1$ pendant vertices, and from (1) we have

$$\kappa\bigl(G '\bigr)\le x^{T}Q\bigl(G ' \bigr)x=x^{T}Q(G)x=\kappa(G). $$

If $\kappa(G ')=\kappa(G)$, then $x=(x_{1}, x_{2}, \ldots, x_{n})^{T}$ is also an eigenvector corresponding to $\kappa(G ')$. By Lemmas 2.4 and 2.3, we have $\vert x_{2}\vert >\vert x_{1}\vert >0$, a contradiction. Therefore $\kappa(G ')<\kappa(G)$. By Lemma 2.7, we have $\kappa(U_{n}^{ k+1}(3))\le\kappa(G ')$. It follows that

$$\kappa\bigl(U_{n}^{ \Delta-1}(3)\bigr)=\kappa\bigl(U_{n}^{ k+1}(3) \bigr)< \kappa(G). $$

Now we assume that $g=3$. If $G\neq U_{n}^{*}(3, \Delta)$, then there are two paths attached at the vertex $v_{1}$ with length more than 1. Without loss of generality, we may assume that $i_{1}\ge3$ and $i_{2}\ge3$. Let $P_{i_{1}}=v_{1}\ldots v_{b}v_{a}$ and $P_{i_{2}}=v _{1}\ldots v_{d}v_{c}$. Without loss of generality, we may assume that $\vert x_{b}\vert \ge \vert x_{d}\vert >0$. Let $G '=G-v_{d}v_{c}+v_{b}v_{c}$. Then $\Delta(G ')=\Delta$, $G '$ has $k+1$ pendant vertices. By Lemma 2.5, we have $\kappa(G ')<\kappa(G)$. It follows from Lemma 2.7 that

$$\kappa\bigl(U_{n}^{ \Delta-1}(3)\bigr)=\kappa\bigl(U_{n}^{ k+1}(3) \bigr)\le\kappa\bigl(G '\bigr)< \kappa(G). $$

If $G=U_{n}^{*}(3, \Delta)$ and $\Delta\le n-5$, by Lemma 2.3, we have $\vert x_{n-4}\vert >\vert x_{3}\vert $. Let

$$G_{1}=U_{n}^{*}(3, \Delta)-v_{3}v_{\Delta}+v_{n-4}v_{\Delta}. $$

Let $s=\Delta-4$. Then by Lemma 2.5, we have $\kappa(G_{1})< \kappa(U_{n}^{*}(3, \Delta))$. Let

$$G_{2}=G_{1}-v_{n-4}v_{\Delta}-v_{n-1}v_{n}+v_{n-3}v_{\Delta}+v_{n-2}v _{n}. $$

By Lemma 2.10, we have $\kappa(G_{2})<\kappa(G_{1})$. Noting that $G_{2}$ has $\Delta-1$ pendant vertices, by Lemma 2.7, we have

$$\kappa\bigl(U_{n}^{ \Delta-1}(3)\bigr)\le\kappa(G_{2})< \kappa(G_{1})< \kappa(G). $$

If $G=U_{n}^{*}(3, n-4)$ or $U_{n}^{*}(3, n-3)$, by Lemma 2.11, we have

$$\begin{aligned}& \kappa\bigl(U_{n}^{n-5}(3)\bigr)< \kappa\bigl(U_{n}^{*}(3, n-4)\bigr)=\kappa(G), \\& \kappa\bigl(U_{n}^{n-4}(3)\bigr)< \kappa \bigl(U_{n}^{*}(3, n-3)\bigr)=\kappa(G). \end{aligned}$$

This completes the proof. □

Secondly, we determine the graph whose least signless Laplacian eigenvalue attains the minimum among all graphs in $\mathcal{G}(n, \Delta)$.

### Theorem 3.2

*Let* $4\le\Delta\le n-3$. *Among all graphs in* $\mathcal{G}(n, \Delta)$, $U_{n}^{ \Delta-1}(3)$ *is the unique graph whose least signless Laplacian eigenvalue attains the minimum*.

### Proof

Let $G\in\mathcal{G}(n, \Delta)$ such that $\kappa(G)$ as small as possible, and let $v\in V(G)$ such that $d_{G}(v)=\Delta$. By deleting edges from *G*, we can get a non-bipartite unicyclic spanning subgraph of *G*, denoted by $G'$, such that $d_{G'}(v)=\Delta$. By Lemma 2.1, we have $\kappa(G')\leq\kappa(G)$. By Theorem 3.1, we have $\kappa(U_{n}^{ \Delta-1}(3))\le\kappa(G')$ with equality if and only if $G'=U_{n}^{ \Delta-1}(3)$. Therefore

$$\kappa\bigl(U_{n}^{ \Delta-1}(3)\bigr)\le\kappa \bigl(G'\bigr)\le\kappa(G). $$

Noting that *G* is the graph whose least signless Laplacian eigenvalue attains the minimum among all graphs in $\mathcal{G}(n, \Delta)$, we have $\kappa(G)\le\kappa(U_{n}^{ \Delta-1}(3))$. It follows that $\kappa(U_{n}^{ \Delta-1}(3))=\kappa(G)$. This implies that *G* may be obtained from $U_{n}^{ \Delta-1}(3)$ by adding edges. Let $x=(x_{1}, x_{2}, \ldots, x_{n})^{T}$ be a unit eigenvector corresponding to $\kappa(G)$. Then

$$\begin{aligned} \kappa(G) =& \sum_{uv \in E(G)}\bigl(x(u)+x(v) \bigr)^{2} \\ =& \sum_{uv \in E(U_{n}^{ \Delta-1}(3))}\bigl(x(u)+x(v) \bigr)^{2}+ \sum_{uv \in E(G)\setminus E(U_{n}^{ \Delta-1}(3))}\bigl(x(u)+x(v) \bigr)^{2} \\ \ge&\sum_{uv \in E(U_{n}^{ \Delta-1}(3))}\bigl(x(u)+x(v)\bigr)^{2}\ge \kappa \bigl(U_{n}^{ \Delta-1}(3)\bigr). \end{aligned}$$

Since $\kappa(G)=\kappa(U_{n}^{ \Delta-1}(3))$, it follows that

$$\sum_{uv \in E(G)\setminus E(U_{n}^{ \Delta-1}(3))}\bigl(x(u)+x(v)\bigr)^{2}=0,\qquad \sum_{uv \in E(U_{n}^{ \Delta-1}(3))}\bigl(x(u)+x(v)\bigr)^{2}=\kappa \bigl(U_{n} ^{ \Delta-1}(3)\bigr). $$

Therefore $x=(x_{1},x_{2},\ldots, x_{n})^{T}$ is also an eigenvector corresponding to $\kappa(U_{n}^{ \Delta-1}(3))$. By Lemmas 2.4 and 2.3, we have

$$\vert x_{1}\vert =\vert x_{2}\vert \le \vert x_{3}\vert < \vert x_{4}\vert < \cdots< \vert x_{n-\Delta+2}\vert =\cdots= \vert x_{n}\vert . $$

From the eigenvalue equation $Q(U_{n}^{ \Delta-1}(3))x=\kappa(U _{n}^{ \Delta-1}(3))x$, we have $x_{n-\Delta+2}=\cdots=x_{n}$. If $E(G)\setminus E(U_{n}^{ \Delta-1}(3))\neq\emptyset$, then

$$\sum_{uv \in E(G)\setminus E(U_{n}^{ \Delta-1}(3))}\bigl(x(u)+x(v)\bigr)^{2} \neq0, $$

which yields a contradiction. So $E(G)\setminus E(U_{n}^{ \Delta-1}(3))= \emptyset$. Therefore, $G=U_{n}^{ \Delta-1}(3)$. □

### Remark 3.3

For $\Delta=2$, we know that $\mathcal{G}(n, 2)=\{C_{n}\}$ with *n* being odd. For $\Delta=3$, from [4], we know that $U_{n}^{ 1}(3)$ is the unique graph whose least signless Laplacian eigenvalue attains the minimum among all graphs in $\mathcal{G}(n, 3)$. For $\Delta=n-1$, $\mathcal{U}(n, n-1)=\{S_{n}^{*}\}$, where $S_{n}^{*}$ is obtained by adding one edge to the star $K_{1, n-1}$. Let $G\in\mathcal{G}(n, n-1)\setminus\{S_{n}^{*}\}$, then *G* is obtained from $S_{n}^{*}$ by adding at least one edge. By a similar reasoning to that of Theorem 3.2, we can show that $\kappa(S_{n}^{*})< \kappa(G)$. For $\Delta=n-2$, $\mathcal{U}(n, n-2)=\{ S_{n-1}^{*+1}, S_{n-1}^{*+2} \}$, where $S_{n-1}^{*+1}$ is obtained from $S_{n-1}^{*}$ by adding one pendant edge to a vertex of degree 1, and $S_{n-1}^{*+2}$ is obtained from $S_{n-1}^{*}$ by adding one pendant edge to a vertex of degree 2. From Lemmas 2.5, 2.4 and 2.6, we may obtain $\kappa(S_{n-1}^{*+1})<\kappa(S_{n-1} ^{*+2})$. Let $G\in\mathcal{G}(n, n-2)\setminus\{ S_{n-1}^{*+1}, S_{n-1}^{*+2} \}$, then *G* is obtained from $S_{n-1}^{*+1}$ or $S_{n-1}^{*+2}$ by adding at least one edge. By a similar reasoning to that of Theorem 3.2, we can show that $\kappa(S_{n-1}^{*+1})<\kappa (G)$.

## Discussion

Recalling that $\kappa(G)$ depends more on the distribution of the edges of a graph than on their number, we propose the following problems for further research.

1. Characterize all extremal graphs whose least signless Laplacian eigenvalue attains the minimum among all non-bipartite unicyclic graphs with a given degree sequence.
2. Characterize all extremal graphs whose least signless Laplacian eigenvalue attains the minimum among all non-bipartite connected graphs with a given degree sequence.
